# Considering clonal hematopoiesis of indeterminate potential in space radiation risk analysis for hematologic cancers and cardiovascular disease

**DOI:** 10.1038/s43856-023-00408-4

**Published:** 2024-06-11

**Authors:** Charles M. Werneth, Zarana S. Patel, Moriah S. Thompson, Steve R. Blattnig, Janice L. Huff

**Affiliations:** 1 NASA Langley Research Center, Hampton, VA USA; 2 Center for Scientific Review, National Institutes of Health, Bethesda, MD USA; 3 NASA Lyndon B. Johnson Space Center, Houston, TX USA

**Keywords:** Haematological cancer, Cardiovascular biology

## Abstract

**Background:**

Expanding human presence in space through long-duration exploration missions and commercial space operations warrants improvements in approaches for quantifying crew space radiation health risks. Currently, risk assessment models for radiogenic cancer and cardiovascular disease consider age, sex, and tobacco use, but do not incorporate other modifiable (e.g., body weight, physical activity, diet, environment) and non-modifiable individual risk factors (e.g., genetics, medical history, race/ethnicity, family history) that may greatly influence crew health both in-mission and long-term. For example, clonal hematopoiesis of indeterminate potential (CHIP) is a relatively common age-related condition that is an emerging risk factor for a variety of diseases including cardiovascular disease and cancer. CHIP carrier status may therefore exacerbate health risks associated with space radiation exposure.

**Methods:**

In the present study, published CHIP hazard ratios were used to modify background hazard rates for coronary heart disease, stroke, and hematologic cancers in the National Aeronautics and Space Administration space radiation risk assessment model. The risk of radiation exposure-induced death for these endpoints was projected for a future Mars exploration mission scenario.

**Results:**

Here we show appreciable increases in the lifetime risk of exposure-induced death for hematologic malignancies, coronary heart disease, and stroke, which are observed as a function of age after radiation exposure for male and female crew members that are directly attributable to the elevated health risks for CHIP carriers.

**Conclusions:**

We discuss the importance of evaluating individual risk factors such as CHIP as part of a comprehensive space radiation risk assessment strategy aimed at effective risk communication and disease surveillance for astronauts embarking on future exploration missions.

## Plain language summary

Space radiation exposure is a major hazard of spaceflight that may increase cancer and cardiovascular disease risks for future astronauts exploring the moon and Mars. There is a need for accurate risk assessment that considers individual risk factors to support informed consent and medical management of these risks. Clonal hematopoiesis of indeterminate potential (CHIP) is a condition that occurs when copies of variant cells accumulate in the blood of otherwise healthy individuals. CHIP is an emerging risk factor linked with blood cancers and cardiovascular disease. We evaluated how CHIP can alter space radiation health risks in astronauts for a Mars exploration mission scenario. We find large increases in lifetime risk of space radiation exposure-induced death for hematologic malignancies and cardiovascular disease in CHIP carriers. These results suggest that increased screening may help facilitate better management of radiation risks.

## Abstract (web summary)

Werneth et al. evaluate the impact of clonal hematopoiesis of indeterminate potential (CHIP) on health risks associated with space radiation exposures in astronauts. This work highlights the importance of considering personalized risk factors, such as CHIP, for effective risk assessment and monitoring of astronaut health.

## Introduction

Astronauts are engaged in extended-duration space missions on the International Space Station and are preparing for deep space missions beyond low-Earth orbit as exploration extends farther into the solar system. Among the many hazards experienced by crew members is space radiation exposure, which is composed of ionizing radiation from solar particle events (SPE), galactic cosmic rays (GCR), and trapped particle radiation confined to the Earth’s Van Allen radiation belts. SPE are sporadic and comprise mostly protons with exposures that may be effectively mitigated when crew members are given sufficient time to prepare storm shelters. GCR consist of high mass and energy (HZE) ions—protons and heavier nuclei—that may be highly penetrating to spacecraft shielding and tissue and pose one of the most serious threats to astronaut health both in-mission and post-flight^1^. Heavy ions differ from terrestrial forms of radiation, such as x-rays or gamma-rays, and impart unique biological damage as they traverse tissue and cells. X-rays and gamma-rays are considered low linear energy transfer (LET) forms of radiation and are sparsely ionizing, while heavy ions are considered high-LET and produce distinct tracks of clustered damage as they pass through biological material. Two main categories of health risks associated with space radiation exposure are cancers and cardiovascular disease^2^.

In addition to space radiation, other hazards associated with spaceflight include microgravity, hostile/closed environments, distance from Earth, and isolation and confinement^1^. The full extent of the impacts on human health associated with these combined hazards remains uncertain; however, there are a range of common molecular changes identified through studies conducted in astronauts and in model organisms. These include complex DNA damage, altered levels of oxidative stress, mitochondrial dysfunction, epigenetic modifications, telomere length alterations and microbiome changes^3^. For example, complex cytogenetic damage was observed in peripheral blood lymphocytes taken from astronauts following missions on the International Space Station^4^. These measurements provide a useful retrospective indicator of radiation dose and are considered a surrogate marker of cancer risk^5,6^. Alterations in telomere length dynamics are also observed during and after spaceflight and are proposed as a useful method to evaluate individual crew member response to the harsh environment in space^7^.

To evaluate radiation risks in determining astronaut flight assignments, the National Aeronautics and Space Administration (NASA) space cancer risk model (NSCR) is used to calculate excess lifetime risks of solid and hematologic cancer incidence (risk of exposure-induced cancer or cases; REIC) and mortality (risk of exposure-induced death; REID), which are based on average male and female United States (U.S.) population disease rates for never-smokers^8–10^. The NSCR also includes the capability to calculate REID for stroke and coronary heart disease (CHD), based on average U.S. population disease rates^11,12^. However, there are inherent uncertainties associated with using population averages that limit their applicability to individuals, especially healthy individuals such as astronauts. Consideration of additional modifiable and non-modifiable risk factors is also important in determining the likelihood that an individual will develop adverse outcomes associated with radiation exposure^13–15^. Modifiable risk factors are features that can be controlled or altered, whereas non-modifiable risk factors cannot be changed or controlled. Cardiovascular disease (CVD) modifiable risk factors include smoking, obesity, unhealthy diet, lack of physical activity, high blood pressure and cholesterol levels^16^. Non-modifiable risk factors include sex, age, genetics, ethnicity/race, family and personal medical history. Many of these are also risk factors for cancer^17^. Risk prediction models that incorporate these types of risk factors are used in clinical settings for personalized prevention in cardiovascular health and for certain cancers^18–21^. Examples include the well-known Framingham risk score, which was the first CVD risk prediction tool, and the Gail model/Breast Cancer Risk Assessment Tool for the prediction of a woman’s risk of developing invasive breast cancer^18–23^. These tools can be incorporated into a comprehensive radiation risk assessment strategy for space crews that extends modeling capability to include informed consent and medical management of space radiation-related health risks^15,24^.

Evaluation of emerging risk factors that could have a substantial impact on mission-related health risks is also very important. Clonal hematopoiesis of indeterminate potential (CHIP) is a developing risk factor with a fairly high population prevalence that exhibits morbidity and mortality outcomes aligning with the major health risks associated with space radiation exposure, namely cancers and cardiovascular disease. CHIP carriers are asymptomatic yet have clonally expanded hematopoietic stem or early progenitor cells that contain somatic mutations frequently identified as driver mutations associated with myeloid malignancies^25–27^. The most common mutations in CHIP carriers are in genes for the epigenetic regulators (*DNMT3A, TET2, ASXL1*), the signaling protein *JAK2*, the DNA damage response regulators (*TP53, PPM1D)*, and spliceosome components (*SF3B1, SRSF2*)^28^. CHIP prevalence, generally defined as a variant allele frequency ≥2%^29^, is age dependent, increasing over time and present in approximately 10–20% of the population >70 years of age, as detected by whole-genome or whole-exome sequencing^25–27^. Using error-corrected, targeted DNA sequencing, a next-generation sequencing technique with higher sensitivity, clonal hematopoiesis at VAF lower than the 2% CHIP classification threshold can actually be found in nearly all people over the age of 50, including members of the astronaut corps^30–32^. Although CHIP is not associated with clinical symptoms, individual carriers exhibit background (no radiation exposure) risks of hematologic malignancies that are an order of magnitude or higher than those without CHIP^25–27^. For this subpopulation of individuals, the absolute risk of developing hematologic malignancies, mainly of myeloid lineage, is estimated at 0.5–1% per year^25,26,29^. Based on these risk estimates, a crew member with CHIP would have an inherent, albeit small, absolute risk of developing a hematologic cancer during a mission, which may be higher or lower depending on a variety of factors including size of the clone(s), the specific mutation(s), the number of mutations, and astronaut age. CHIP carriers also exhibit a two-fold or greater risk for incident coronary heart disease, ischemic stroke, and increased risks for solid cancers, all-cause mortality, and for developing therapy-related myeloid malignancies^26,33–36^. NASA periodically solicits astronaut candidates from the general population with diverse demographic backgrounds, where crew selection ages have historically spanned 26–46 years of age^37^. Although the mean age of the first flight is 41 years, it remains unclear if future crew selections will follow similar age trends. Many astronauts in the active corps also have previous flight experience, which is currently mostly attributed to flight time spent on the International Space Station. Consequently, future Mars mission crew members will likely be of diverse ages with varying previous flight experience. Also, as the commercial space industry continues to expand, risk modifiers in older populations, and perhaps populations less healthy than astronauts, become increasingly important in informing risk.

To the best of our knowledge, there have been two studies assessing clonal hematopoiesis in astronauts. The first study (Mencia-Trinchant et al.^38^) evaluated samples from identical twin astronauts: one who spent a year on the ISS while the other remained on Earth. Clonal hematopoiesis was detected in both twins at ages younger than would normally be detected. The second study (Brojakowska et al.^30^) revealed somatic mutations in CHIP driver genes, detected retrospectively, in peripheral blood DNA samples from 14 otherwise healthy members of the astronaut corps who had previously flown shuttle missions. In this study, high-sensitivity, error-corrected, targeted DNA sequencing was used, which identified clonal mutations in all samples, albeit at small clone sizes ranging from 0.10 to 0.95%. CHIP therefore represents an important factor that could influence individual radiation sensitivity with both in-mission and post-mission health consequences, highlighting the importance of incorporating personalized strategies in primary prevention, clinical care and disease surveillance for spaceflight crews^13–15,39^. There are many open questions regarding how space radiation and other spaceflight hazards, such as microgravity, may modify clonal dynamics. Longitudinal studies with a larger astronaut cohort alongside further ground-based epidemiology and experimental studies will be needed to draw firm conclusions on connections between CHIP and health risks associated with space radiation and spaceflight. However, using current NASA models and CHIP risk data from published population-based cohort studies^26^, it is possible to evaluate both in-mission and lifetime risks of radiation exposure associated with CHIP carrier status, which is the focus of the current study.

Recently, NASA updated the permissible exposure limit (PEL) for space radiation, transitioning from a risk-based limit to one based on lifetime effective dose. The new limit is currently set at 600 mSv, which ensures that astronaut space radiation exposure remains below a mean value of 3% REID for radiogenic cancers, calculated with the NSCR model^9,10,40^. The new limit is applicable to both male and female crew members irrespective of age and is used operationally in astronaut certification for flight^10,40^. The new permissible exposure limit ensures equality across the corps for crew flight assignments; however, it is also associated with a large increase in accepted radiation-induced health risks^41^. The acceptance of greater risk puts an increased emphasis on the need for new knowledge and tools to support accurate risk assessment, informed consent for astronaut crews, and approaches for personalized medical management of these health risks^14,15,39^.

Space radiation exposure estimates differ from typical terrestrial exposures received during medical diagnostic imaging and cancer radiotherapy. The average effective dose is 0.02 mSv for a chest x-ray and 7 mSv for a computed tomography scan of the chest^42^, whereas cancer radiotherapy exposures to tumors are on the order of 20 Gy or higher^43^. Space exposures vary greatly and depend on many factors, including shielding configurations, orbital constraints, time during the solar cycle, and duration in deep space during transit. NASA has aggregated several Mars mission scenarios, known as design reference missions (DRMs), which span from short transit times in deep space with short duration stays on the Martian surface to much longer missions that include both long transit times and surface visits. Effective dose estimates are as follows^2,44,45^: low-Earth-orbit mission (6–12 months): 50–200 mSv; lunar surface mission (42 days): 70 mSv; sustained lunar operations (12 months): 300–400 mSv; Mars missions (16–30 month): 400–1200 mSv.

The present study considers a scenario representative of potential, future astronaut crews who will embark on Mars missions, chosen to represent a cohort who are at higher risk for CHIP. The authors use data from a human cohort study by Jaiswal et al.^26^ to show that CHIP carriers are at much greater risk of exposure-induced death as compared to the average background U.S. population of never-smokers for a Mars mission scenario.

## Methods

The present section describes key components of the NSCR that were modified to incorporate CHIP hazard ratios (HRs) into the background hazard rates for CHD, HC, and stroke mortality. First, space radiation and nuclear transport are described, followed by a description of the NSCR model components, and the modifications required to incorporate HRs from a human cohort study of CHIP.

### Space radiation environment and radiation transport

The space radiation environment is composed of ionizing radiation from galactic cosmic rays (GCRs), solar particle events (SPEs), and geomagnetically trapped particles^46,47^. GCRs are formed from the shock waves of supernovae and consist of protons, alpha particles, and heavier nuclei with energies that may approach the speed of light^46,47^. The omnipresent GCR fluence in space is dependent on the solar cycle and receives the greatest attenuation during solar maximum^47^. GCRs may be highly penetrating and pose considerable radiation risk for crew members on long-duration missions beyond low-Earth orbit, such as deep space and Mars missions. GCR exposures are qualitatively different from the gamma- and x-ray exposure typically experienced in radiotherapy and medical scans on Earth. GCR exposure is also a concern for the upcoming Artemis lunar missions; although the early missions will be relatively short, exposures will be small.

SPEs are sporadic events that occur more frequently during solar maximum and are primarily composed of protons. Because of their unpredictable nature, SPEs present a risk for all mission scenarios, though with sufficient warning, crew members may erect protective radiation shelters to partially mitigate the radiation exposure^48^. Finally, during low-Earth orbit missions (such as missions on the International Space Station), crew members are exposed to radiation that has become confined within the geomagnetic field of the Earth^49^.

The intensity of the GCR spectrum varies with the 11-year solar cycle and is characterized by greater solar activity during solar maximum and lower activity during solar minimum. The GCR flux is most effectively attenuated during the apex of the solar cycle, when the solar magnetic field strength is greatest, whereas the least attenuation of the GCR flux occurs at solar minimum^47^. The GCR flux may be mitigated by an order of magnitude or more during solar maximum, which results in lower overall mission exposures, although there is an increased propensity for SPEs to occur. It is expected that most crew exposure will be accumulated during transit in deep space to Mars. As a result, the 2001 GCR flux at solar maximum and the historical August 1972 SPE are often employed in trade studies for risk estimates of Mars missions^50,51^. The large variation in fluxes between solar cycles leads to effective doses at solar minimum that are approximately twice as large as those at solar maximum.

Projectile particles from the space radiation environment may collide with nuclei in shielding materials and yield projectile and target (shield) fragmentation products. Additional interactions may occur as the projectile and target constituents propagate through the shielding materials, which leads to a complex field of radiation to which crew members are exposed. The nuclear interactions are described by models of nuclear cross sections^52–54^, and the propagation of the incident radiation and all of the fragmentation products produced during the internuclear cascade is described by the Boltzmann radiation transport equation. NASA uses the deterministic radiation transport code, High Charge and Energy TRaNsport (HZETRN)^54–56^, for radiation transport because of its high efficiency (in comparison to Monte Carlo codes), which is required for estimating accurate particle fluences in realistic vehicles. Male Adult Voxel^57^ and Female Adult Voxel^58^ human phantom body models are used as surrogates for human tissue. Particle fluences from HZETRN are then used to evaluate dosimetric quantities—such as the dose, dose equivalent, effective dose—and the REID.

### NASA space cancer risk model

The NSCR projects the risk of exposure-induced death and consists of two main components: a risk transfer model based on terrestrial radiation epidemiology and a model that scales from low linear energy transfer (LET) terrestrial radiation exposures to high-LET space radiation exposures. A major challenge with space radiation is the dearth of high-LET human data to inform risk models due to the small cohort of astronauts. Consequently, NASA utilizes low-LET gamma-ray exposure risk models from the Japanese atomic bomb cohort Life Span Studies (LSS)^59,60^ and implements a scaling procedure to estimate space radiation risk. The LSS study has developed excess relative risk (ERR) and excess absolute risk (EAR) models of cancer incidence and mortality by comparing the disease rates of the individuals located within 3 km of atomic blast hypocenter to unexposed persons beyond the 3 km radius. The ERR is proportional to the background cancer mortality or incidence rates, and the EAR is independent of the background cancer mortality rates. These risk models are transferred to the population of interest, which, in the present study, is the average U.S. population of never-smokers.

The inhabitants of Hiroshima and Nagasaki were exposed to radiation delivered at high dose rates, but the total dose was highly dependent on the distance from the hypocenter and any shielding provided by buildings where the exposed individuals may have been located^61^. Chronic exposure from cosmic rays is delivered at lower dose rates, and total mission doses depend on mission duration, location, shielding conditions, and other factors. Experimental data for cancer surrogates obtained at the NASA Space Radiation Laboratory are used to develop the radiation quality factor, *Q*, that scales from low-LET to high-LET exposure doses. The dose and dose-rate effectiveness factor (DDREF) translates the low-LET risk models to dose rates more appropriate for space radiation exposures, where a linear no-threshold model of cancer induction from radiation is assumed.

The REID for specific endpoint (for example, HC, stroke, colorectal cancer, etc.) *T*, is given by^9,62,63^

1 $${\rm REID}_{T} ={\int }_{{a}_{E}}^{{a}_{\max }}{\lambda }_{T}\left(a,{a}_{E},{w}_{T},{H}_{T},{\delta }_{T}\right){S}_{0}\!\left(a|{a}_{E}\right){e}^{-{\sum}_{T'} {\int }_{{a}_{E}}^{a}{\lambda }_{T'}(t,{a}_{E},{w}_{T'},{H}_{T'},{\delta }_{T'}){dt}}{da},$$

where *λ*_*T*_(*a*, *a*_*E*_, *w*_*T*_, *H*_*T*_, *δ*_*T*_) is the mortality hazard rate for a specific solid cancer, CHD, or stroke; *a* is the attained age; *a*_*E*_ is the exposure age; *w*_*T*_ are the tissue weighting factors; *H*_*T*_ is the tissue dose equivalent; *δ*_*T*_ is the dose and dose-rate effectiveness factor; *da* is the differential age element; *S*_0_(*a* | *a*_*E*_) = *S*(*a*)/*S*(*a*_*E*_) is the conditional probability of survival of the background population; and the exponential term accounts for competing causes of death due to radiation exposure.

NASA uses the low-LET excess cancer risk models from the LSS study of the Japanese atomic bomb cohort for gamma-ray exposure, where the high dose rates of the atomic bomb cohort are scaled to the moderately low dose rates of space by the DDREF. The cancer hazard rate is a mixture of ERR and the EAR models:

2 $${\lambda }_{T}\left(a,{a}_{E},{w}_{T},{H}_{T},{\delta }_{T}\right)= \, {w}_{T}{{{\mbox{ERR}}}}_{T}\left(a,{a}_{E},{H}_{T},{\delta }_{T}\right){\lambda }_{0,T}\left(a\right)\\ +\left(1-{w}_{T}\right){{{\mbox{EAR}}}}_{T}\left({a,a}_{E},{H}_{T},{\delta }_{T}\right),$$

where the ERR is proportional to the background population hazard rate, *λ*_0,*T*_(*t*), and the EAR is independent of *λ*_0,*T*_(*t*). The ERR model is preferred  for most tissues with *w*_*T*_ = 0.7, whereas smaller ERR weights (which correspond to higher EAR weights) are preferred for lungs (*w*_*T*_ = 0.5), HC (*w*_*T*_ = 0.5), and breast (*w*_*T*_ = 0). The tissue-specific dose–response models are from the United Nations Scientific Committee Reports^64^, Biological Effects of Ionizing Radiation^60^, and from studies by Preston et al.^65^. The NSCR  utilizes low-LET risk models that are based on incidence for all tissues with the exception of HC models, which are based on cancer mortality data.

The tissue dose equivalent is related to the NASA radiation quality factor, *Q*_NASA_, by

3 $${H}_{T}=\frac{1}{\rho }\frac{1{0}^{8}}{6.24}\mathop{\sum }_{A}\mathop{\sum }_{Z}\int {\varphi }_{T}(E,A,Z)L{Q}_{{NASA}}{dE}.$$

*φ*_*T*_ is the differential fluence (particles/[cm^2^-MeV/n], *L* is the LET (keV/µm), *A* is the mass, *Z* is the charge, *E* is the kinetic energy (MeV/n), and *ρ* is the material bulk density (1.1 g/cm^3^) in tissue, and *Q*_NASA_ is the NASA radiation quality factor^9^.

Estimates of circulatory disease from low-level ionizing radiation have been incorporated into the NSCR model. Little et al.^12^ performed a systematic review and meta-analysis of peer-reviewed manuscripts of human circulatory disease mortality and morbidity. ERR models of CHD and ischemic stroke were fit to the available data and were insensitive to age after radiation exposure. These models are used to scale background mortality rates in the NSCR.

Uncertainties associated with particle fluence, radiation quality, and low-LET radiation risk models are represented with probability distribution functions, which are propagated through the NSCR model with Monte Carlo sampling techniques. The total REID is obtained by summing over the REID% distributions for CHD, stroke, HC, and other solid cancers tracked in the NSCR, where risk projections are expressed as statistical measures, such as the median, mean, and confidence limits of the corresponding total REID distributions. Moreover, optimal values of the uncertainty distributions may be used to find the point estimate of the REID.

### Human cohort study of CHIP

The recent study by Jaiswal et al.^26^ revealed increased risks of incident CHD, ischemic stroke, and all-cause mortality for CHIP carriers when compared to people without the characteristic genetic mutations associated with CHIP. Whole-exome sequencing of DNA in peripheral blood cells was analyzed in 17,182 people with a median age of 58 years, where the sequences were screened for 160 genetic mutations that are typically associated with hematological cancers. The detection limit for single nucleotide variants was at a variant allele fraction 3.5%, whereas the detection for indels was 7%. The study revealed that over 800 individuals had acquired mutations in one or more of 73 genes associated with hematological disease. The most frequent genetic mutations were found in *DNMT3A*, *TET2*, and *ASXL1*.

The data from the Jaiswal et al.^26^ study are represented as HRs with corresponding 95% confidence limits. The HR is defined as the ratio of the hazard rates of those with the disease (CHIP) to those without. The hazard rate, *λ*(*t*), is the probability that a hazard occurs to an individual within an infinitesimal time interval (*t,t* + *δt*), provided that the individual is alive at the beginning of that time interval. Therefore, the HR may be expressed as HR = *λ*_*D*_(*t*)/ *λ*_*ND*_(*t*), where *D* is the diseased state and *ND* is the non-diseased state. In the present work, the hazard rate of disease is *λ*_*D*_(*t*) = *λ*_*CHIP*_(*t*), and the non-diseased hazard rate is approximated by the hazard rate of the background population, *λ*_*ND*_(*t*) ≈ *λ*_0*,T*_(*t*). Consequently, the CHIP HR data (HR_*CHIP*_) from the Jaiswal et al.^26^ study may be used to scale the background hazard rates in the NSCR such that *λ*_*CHIP*_(*t*) = HR_*CHIP*_*×λ*_0*,T*_(*t*), where *T* refers to stroke, CHD, and HC. Moreover, the all-cause mortality is incorporated by modifying the conditional probability of survival of the background population^50^.

Jaiswal et al.^26^ found CHIP hazard ratios of HR = 11.1 [3.9, 32.6] for hematologic cancers, HR = 2.0 [1.2, 3.4] for incident CHD, HR = 2.6 [1.4, 4.8] ischemic stroke, and HR = 1.4 [1.1, 1.8] for all-cause mortality, where the values in the brackets are the 95% confidence intervals. To evaluate the REID in Eq. (1), it has been assumed that mortality hazard rates are proportional to incidence rates. The CHIP HR are used to scale the background mortality rates for all ages after radiation exposure. The HR from Jaiswal et al.^26^ for ischemic stroke and hematologic cancers are assumed to apply to stroke (cerebrovascular disease), and HC (malignant neoplasms of lymphatic and hematologic tissue, also referred to as blood-forming organs), respectively, as quantified in the NSCR^9,12,66,67^. Furthermore, probability distribution functions of HR uncertainties are propagated through the NSCR model using Monte Carlo sampling techniques that were employed to include all other uncertainties.

### Statistics and reproducibility

The HZETRN2020 and NSCR codes used for this study are found at https://software.nasa.gov. Characterization of the space radiation environment, spacecraft geometry and composition, and radiation transport were performed with the HZETRN2020 code, and the REID% was evaluated with the NSCR code. REID% distributions were evaluated with Monte Carlo sampling of parameters associated with uncertainty distributions^9^. HZETRN2020 was used to deterministically evaluate the tissue (or endpoint) dose equivalent inside the Male Adult Voxel and Female Adult Voxel human phantoms. A REID distribution for each tissue was obtained by using a Monte Carlo sample size of 100,000, and the total REID was found by summing over all tissue-specific REID distributions. A Monte Carlo sample size of 100,000 ensured that statistical estimates vary by less than 2%. That is, when an additional 100,000 samples are obtained with a different random-number seed, the differences in statistical estimates are no larger than 2%.

In order to verify that the changes to the NSCR were correct, tissues or endpoints that use only age- and sex-independent low-LET ERR models^12^, such as the stroke and CHD excess radiation risks, were modified by a scalar greater than unity from age 50 years until the end of life. Since ERR modes are proportional to background hazard rates, these modifications approximately result in commensurate increases in the tissue-specific REID percent. In contrast, HC is described with a 50% mixture of age- and sex-dependent ERR and EAR models^9^. HR scaling was only applied to the background hazard rates that are proportional to ERR. Consequently, increases in the REID% for HC were expected to be approximately one-half of the scalar modifier at most. Both test cases were verified as correct.

Uncertainties in the HRs were incorporated with Monte Carlo sampling of probability distribution functions that were constructed from the HR data. Jaiswal et al.^26^ reports HRs as *µ* [*α*, *β*], where *µ* was assumed to represent the median, and [*α*, *β*] represents the 95% confidence interval. Uncertainty propagation verification showed that the medians and the confidence intervals were maintained when evaluating the REID. Although HR uncertainties were verified as being properly propagated, the large NASA radiation quality factor uncertainties^68^ tend to mask the other uncertainties in the NSCR, including that of the low-LET risk and physics models.

### Overview of CHIP analysis in the NSCR model

**Fig. 1:**
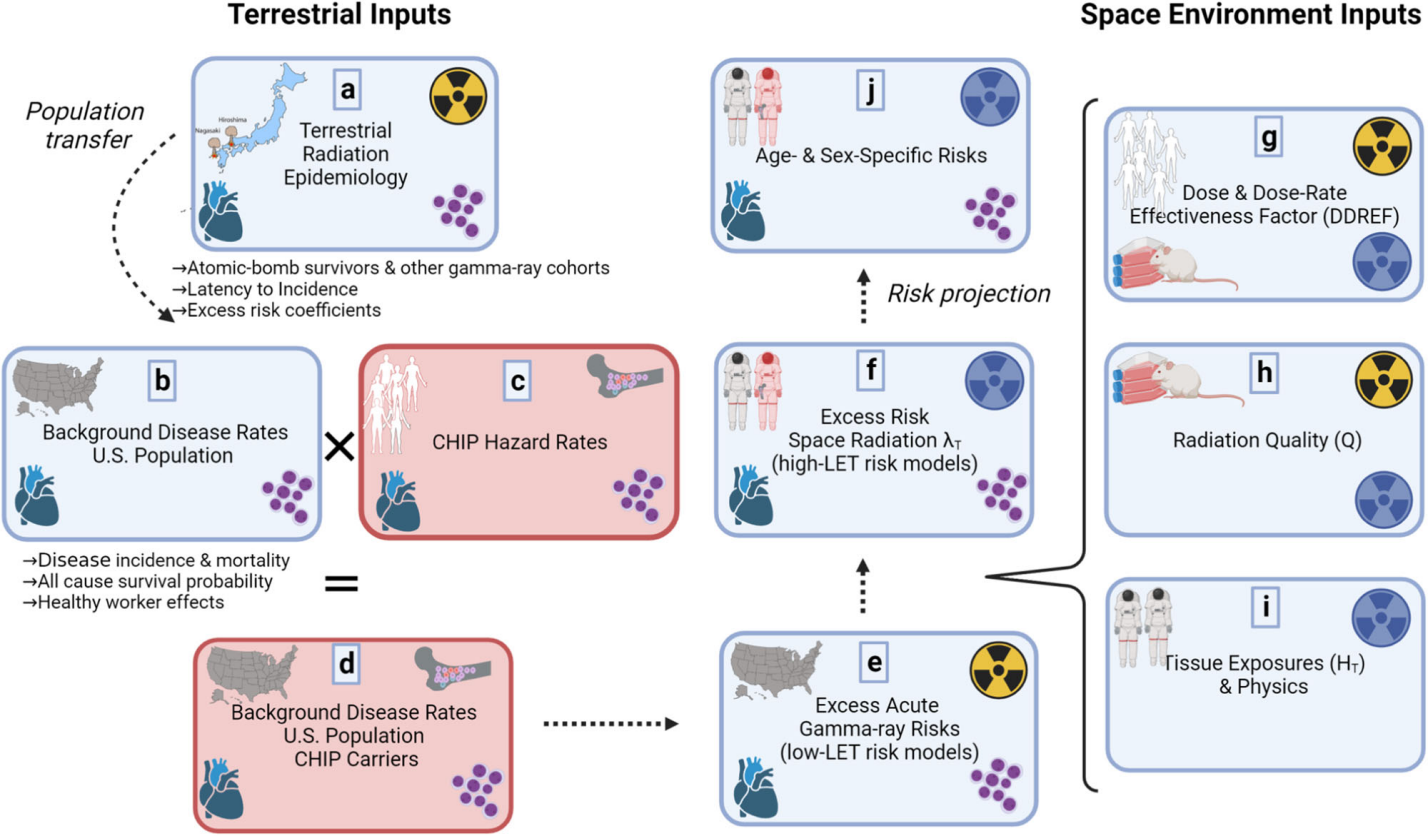
An overview of the NASA Space Cancer Risk model and changes needed to incorporate clonal hematopoiesis of indeterminate potential (CHIP) hazard ratio data. Excess radiation risk models from the Life Span Study of the Japanese atomic bomb cohort (**a**) are transferred to background disease rates of the U.S. population (**b**), which are scaled by CHIP hazard ratio data (**c**) to approximate the background disease rates of average United States. background population of CHIP carriers (**d**). CHIP modifications are incorporated into acute gamma-ray exposure excess risk models (**e**). Space radiation risk (**f**) is estimated by scaling the acute gamma-ray risks by the dose and dose-rate effectiveness factor (**g**) and space radiation dose by the radiation quality factor (**h**) to obtain the tissue dose equivalent (**i**). Space radiation risks are estimated (**j**) by evaluating the excess risk of space radiation (**f**) as a function of the tissue-specific dose equivalent (**i**). The radiation quality factor, *Q*, is used to scale the doses from the space radiation environment to the atomic bomb exposures. The tissue dose equivalent is defined as *H*_*T*_ = *D*_*T*_*Q*, where *D*_*T*_ is the absorbed dose in tissue. Adapted from Simonsen and Slaba^8^ with express permission from the authors. Created with BioRender.com.

A concise overview of the NSCR model and a description of the modifications performed for the present analysis are presented in Fig. 1. Figure 1a represents the excess radiation risk models from the LSS study of the Japanese atomic bomb cohort, which is transferred, according to established procedures, to the population of interest—the disease rates of the average U.S. background population^60,65^, (Fig. 1b). These disease rates are scaled by CHIP HR data (Fig. 1c) to approximate the background disease rates of average U.S. background population of CHIP carriers (Fig. 1d) and are proportional to the ERR model. At this stage the NSCR now includes CHIP modifications for acute gamma-ray exposure (Fig. 1e). The excess space radiation risk (Fig. 1f) is estimated by scaling the acute gamma-ray risks by the DDREF (Fig. 1g) and space radiation dose by the radiation quality factor (Fig. 1h) to obtain the tissue dose equivalent (Fig. 1i). Finally, age- and sex-specific space radiation risks are projected (Fig. 1j) by evaluating the excess risk of space radiation (Fig. 1f) as a function of the tissue-specific dose equivalent (Fig. 1i). A similar approach was used to assess the sparing effect of radioprotective agents on space radiation risks^50,51^.

### Reporting summary

Further information on research design is available in the Nature Portfolio Reporting Summary linked to this article.

## Results

This section examines the impact of incorporating CHIP hazard ratios (HR) from published studies of cohorts ≥50 years of age^26^ for incident heart disease (CHD), cerebrovascular disease, stroke, and hematologic cancers (HC) in the NSCR model by scaling the average background U.S. population mortality rates of never-smokers^9^. The NSCR model projects the sex-specific excess risk of radiation exposure-induced death (REID) from cancer, HC, CHD, and stroke, where uncertainties in radiation quality, low-LET risk coefficients, and particle fluence are propagated by Monte Carlo sampling of probability distributions representative of uncertainty. Point estimates of the REID are found by using the most likely values of uncertainty distribution parameters, and the median, average, and confidence limits are found from statistical analysis of the total REID% distribution. A comprehensive description of the NSCR model and its underlying assumptions are discussed in the Methods section.

The increase in REID is discussed for astronauts exposed to space radiation during a Mars mission scenario. In addition to the end-of-life metric of the REID that is related to the NASA PELs, the REID% is also estimated in 10-year intervals post-radiation exposure to better inform flight surgeons and crew members of risks after the mission, prior to end of life. Point estimates of the REID% are used to obtain HC, CHD, stroke, and solid cancer contributions to the total REID%.

Male and female astronauts are assumed to be exposed at age 50 years to the 2001 solar maximum GCR spectrum^69^ and the August 1972 SPE^70^ during one of the shortest Mars DRMs^45^: 15-month transit (round trip) between Earth and Mars and a 1-month stay on the Martian surface within a 20 g/cm^2^ spherical aluminum shield. The average quality factor as computed at the lung is 3.0, and the average DDREF is 1.5^9^. This mission yields an effective dose of 100 mSv from the SPE and 295 mSv from GCR exposure. The total effective dose for this mission of approximately 395 mSv does not exceed the NASA PEL.

**Fig. 2:**
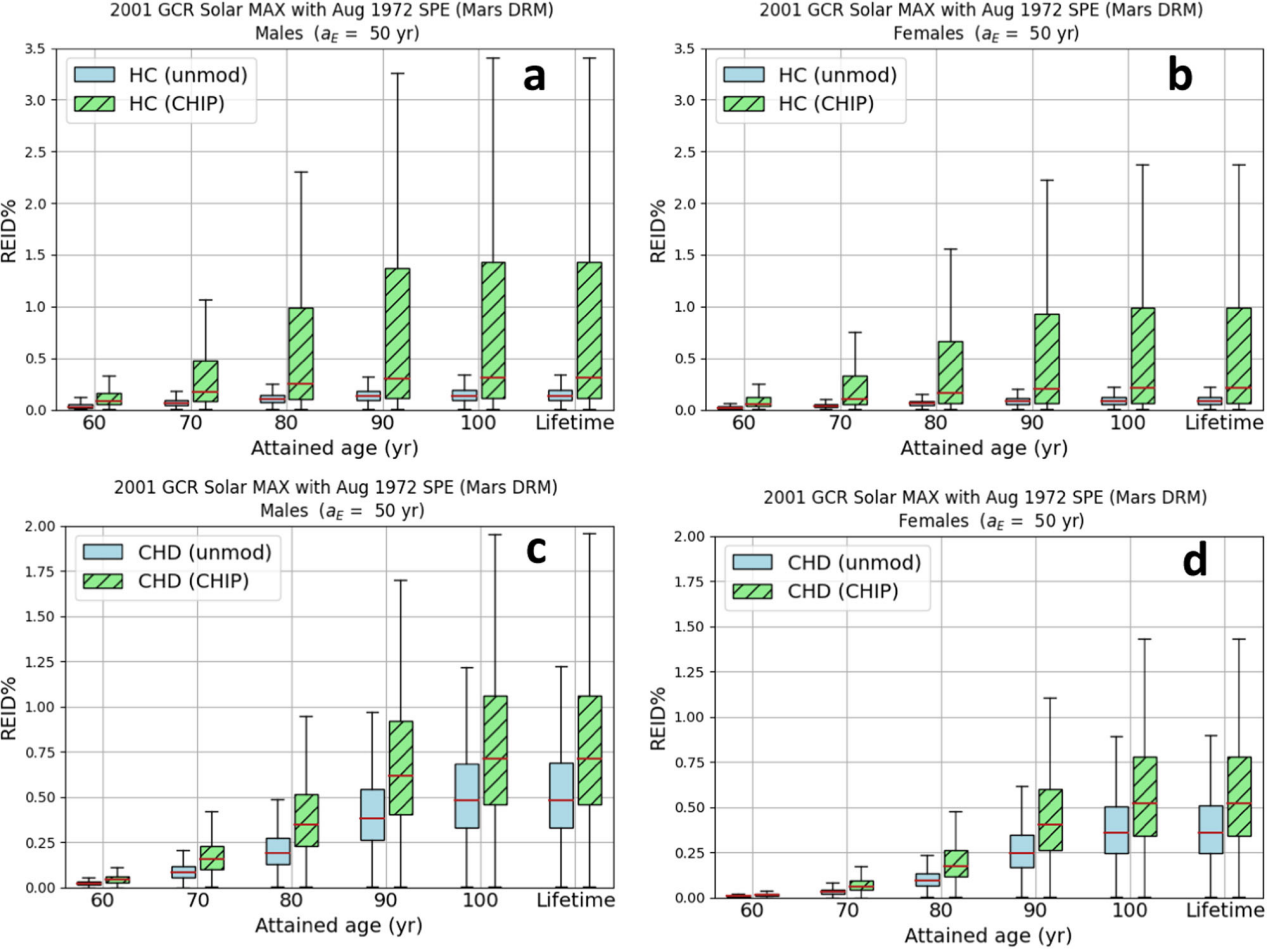
Risk of exposure-induced death % (REID%) for hematologic cancer and coronary heart disease for clonal hematopoiesis of indeterminate potential (CHIP) carriers compared to the average United States background never-smoker population. Hematologic cancer (HC) and coronary heart disease (CHD) risk of exposure-induced death % (REID%) comparisons of the average United States background never-smoker population to CHIP carriers for a Mars design reference mission (DRM) scenario. HC REID% for males (**a**) and females (**b**) and CHD REID% for males (**c**) and females (**d**). Astronauts in a 20 g/cm^2^ spherical aluminum shield are exposed to the 2001 galactic cosmic ray (GCR) at solar maximum (MAX) and the 1972 Solar Particle Event (SPE). Unmodified (unmod) background mortality rates are shown with un-hashed bars, and the CHIP-modified background mortality rates are shown with hashed bars. Uncertainty propagation was implemented with a Monte Carlo sampling size of *N* = 100,000 for each attained age. Median values are shown as red horizontal lines in the box plots. The upper whisker (UW) and lower whisker (LW) of the box plots are defined in terms of the inner quartile range (IQR)—the difference between the  third (Q3) and first (Q1) quartiles: LW = Q_1_ – 1.5×IQR and UW = Q_3_ + 1.5×IQR.

The REID% versus attained age (Fig. 2) is shown for 50-year-old astronauts exposed to the 2001 GCR environment at solar maximum and the August 1972 SPE for the Mars design reference mission. REID% distributions are represented by box plots. The bottom edges of the box plots in Fig. 2 represent the first quartile, Q_1_, (25% of the cumulative distribution function) and the top edges of the box plots represent the third quartile, Q_3_ (75% of the cumulative distribution function). The median of the distribution is indicated with the horizontal solid line in red. The inner quartile range (IQR) is given by IQR = Q_3_ – Q_1_. The lower whisker (LW) and upper whisker (UW) are defined as LW = Q_1_ – 1.5×IQR and UW = Q_3_ + 1.5×IQR. Note that if the lowest datum point is greater than Q_1_   – 1.5×IQR, then LW is set equal to the lowest datum point. Likewise, if the largest datum point is less than Q_3_ + 1.5×IQR, then the upper whisker is set equal to the largest datum point. Increases in the HC (panels a and b) and CHD (panels c and d) REID% are shown for CHIP carriers in comparison to the REID% computed with the unmodified (labeled as unmod in the figures) background mortality rates of the average U.S. population of never-smokers^9^. The CHIP HC hazard ratio, HR = 11.1 [3.9, 32.6], results in large increases of the median and uncertainty. Additional CHIP modifications to the conditional probability of survival and competing causes of radiation-induced death have also been included. The HC REID% for CHIP carriers is smaller for females for all ages after radiation exposure, whereas increases in the CHD REID% are also shown but with much tighter confidence limits. Again, the male CHIP CHD risk is larger than the risk for females.

**Fig. 3:**
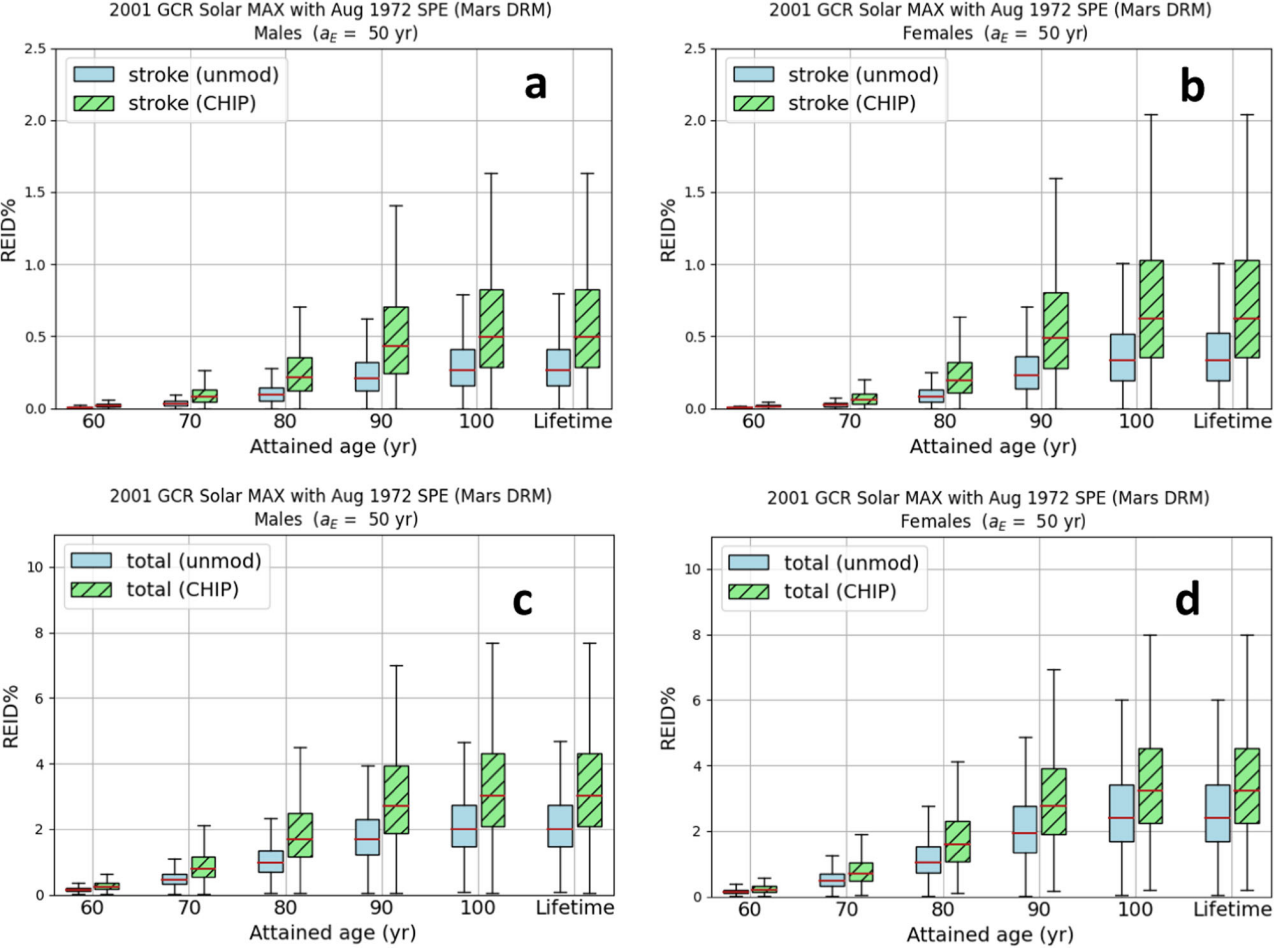
Risk of exposure-induced death % (REID%) for stroke and coronary heart disease for clonal hematopoiesis of indeterminate potential (CHIP) carriers compared to the average United States background never-smoker population. Stroke and total risk of exposure-induced death % (REID%) comparisons of the average United States background never-smoker population to CHIP carriers for a Mars design reference mission (DRM) scenario. Both the background and CHIP populations consist of never-smokers. Stroke REID% for males (**a**) and females (**b**) and total REID% for males (**c**) and females (**d**). Astronauts in a 20 g/cm^2^ spherical aluminum shield are exposed to the 2001 galactic cosmic ray (GCR) at solar maximum (MAX) and the 1972 solar particle event (SPE). Unmodified (unmod) background mortality rates are shown with un-hashed bars, and the CHIP-modified background mortality rates are shown with hashed bars. Uncertainty propagation was implemented with a Monte Carlo sampling size of *N* = 100,000 for each attained age. Median values are shown as red horizontal lines in the box plots. The upper whisker (UW) and lower whisker (LW) of the box plots are defined in terms of the inner quartile range (IQR)—the difference between the third (Q3) and first (Q1) quartiles: LW = Q_1_ – 1.5×IQR and UW = Q_3_ + 1.5×IQR.

The stroke (panels a and b) and total (panels c and d) REID% are shown (Fig. 3) for CHIP carriers in comparison to the REID% projected with the unmodified background mortality rates. Larger REID% increases are observed for females than males, which is likely due to the differences in baseline stroke mortality rates since the excess relative risk (ERR) model for stroke is sex independent. The total REID% (panels c and d) increase associated with CHIP is slightly larger for females than for males. Sex differences in risk may be attributed to other risk factors such as larger baseline risks for females due to radiation-induced lung cancer in the NSCR.

**Fig. 4:**
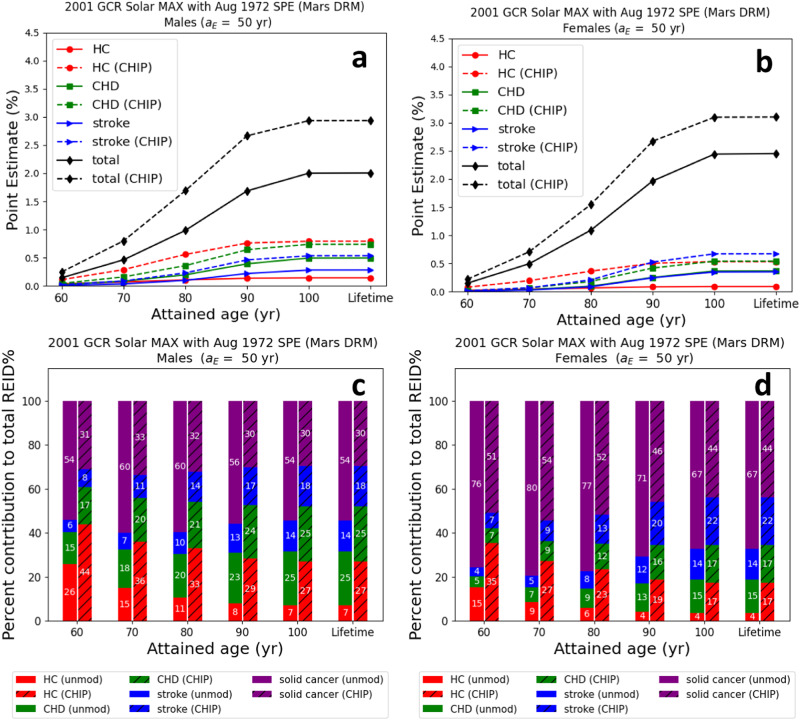
Tissue or endpoint contributions to the total risk of exposure-induced death % (REID%) for clonal hematopoiesis of indeterminate potential (CHIP) carriers compared to the average United States background never-smoker population. Point estimates (**a**, **b**) and percent contribution (**c**, **d**) to the total risk of exposure-induced death (REID) by tissue (or endpoint) of the average United States background never-smoker population compared to clonal hematopoiesis of indeterminate potential (CHIP) carriers for a Mars design reference mission (DRM) scenario. Astronauts in a 20 g/cm^2^ spherical aluminum shield are exposed to the 2001 galactic cosmic ray (GCR) at solar maximum (MAX) and the 1972 Solar Particle Event (SPE). Uncertainty propagation was implemented with a Monte Carlo sampling size of *N* = 100,000 for each attained age, and point estimates of the REID are found by using the most likely values of uncertainty distribution parameters. REID% point estimates of hematologic cancer (HC), coronary heart disease (CHD), stroke, and solid cancers for males (**a**) and females (**b**) and percent contribution to the total REID% for males (**c**) and females (**d**). REID% point estimates (**a**, **b**) for the unmodified (unmod) background are shown with solid lines and symbols, whereas point estimates for the CHIP-modified background are shown with dashed lines and symbols. HC is indicated with red circles; CHD with green squares; stroke with blue triangles; and total with black diamonds. The solid un-hashed bars (**c**, **d**) represent calculations with unmodified background mortality rates, and the hashed bars represent CHIP-modified background mortality rates. In panels (**c**) and (**d**), HC is indicated in red; CHD in green; stroke in blue; and solid cancers in purple.

Sex-specific point estimates of the REID% are found in Fig. 4a, b and are used to compute the HC, CHD, stroke, and solid cancer contributions to the total REID% (panels c and d). The solid cancer contributions include all cancers tracked in the NSCR model, except the HC. The male CHIP carriers have a larger increase in risk than females for all ages after radiation exposure. Also, the combined CHIP risk for males from HC, CHD, and stroke exceeds that of solid cancer risk for all ages after exposure, whereas that trend is observed later in life for females.

Figure 4c, d shows that the solid cancer contribution to the total REID% decreases slowly as a function of age of radiation exposure. The relatively small decrease is attributed to larger contributions to the total REID% from CHD and stroke. The background mortality rates from CHD are greater for men than women, and the stroke risk is larger for women. Since CHD contributes more greatly in men and is a more prevalent risk as compared to stroke, the solid cancer risks for males are smaller; that is, CHD risks are a larger percentage of the total REID% than the female solid cancer risks at the same age after radiation exposure.

Given that the baseline risk of HC for CHIP carriers is greater than the average background population by an order of magnitude^26^, it is prudent to examine the inflight excess radiation risk for the Mars design reference mission. The NSCR utilizes a 2-year step-in latency for HC and 5-year step-in latency for all other tissues, CHD, and stroke. Since the Mars design reference mission studied here is less than 2 years in duration, the inflight REID% is zero. Even when the HC latency is relaxed, the REID% remains small because of insufficient time after radiation exposure to acquire substantial risk.

In contrast to the small inflight excess radiation risks, there is a large increase in the total lifetime REID% for CHIP carriers in comparison to the background population (Table 1). Due to the 11-fold increase of the unirradiated background HC for CHIP carriers, the lifetime HC REID% increases by approximately 400–500%, depending on the mission and exposure conditions. The NSCR uses a 50% mixture of ERR and EAR models for HC, where the background cancer mortality rates are proportional to the ERR. Since HRs were incorporated in the background mortality rates in the present study, the increases of the REID% are expected to be approximately half of the unirradiated background CHIP HC risk. The lifetime CHD, stroke, and total REID% also increase. Since the CHD and stroke hazard rates are sex independent and expressed solely in terms of ERR, the approximately 50% increase for CHD and 90% increase for stroke REID% is expected from HR scaling. The total REID% point estimate increases by 46% for males and 27% for females. Differences in the results may be attributed to the sex-dependent low-LET models for HC and solid cancers. For example, the absolute risk for females (Table 1) is larger than for males because females have a larger radiation-induced lung cancer risk as noted in the LSS studies. Most tissues in the NSCR employ both ERR and EAR models and are sex and age dependent; these differences are manifested in the solid cancers, HC, and total REID%.

**Table 1:** Lifetime risk of exposure-induced death % (REID%) statistics for males and females comparing average United States background never-smoker population to clonal hematopoiesis of indeterminate potential (CHIP) carriers for a Mars mission scenario.

Tissue	PE	Median	Q_1_	Q_3_	IQR	LW	UW
Unmodified background (males)							
HC	1.42 × 10^–^^1^	1.36 × 10^–1^	9.59 × 10^–2^	1.93 × 10^–1^	9.74 × 10^–2^	1.06 × 10^–2^	3.39 × 10^–1^
CHD	4.94 × 10^–1^	4.81 × 10^–1^	3.28 × 10^–1^	6.86 × 10^–1^	3.58 × 10^–1^	1.98 × 10^–3^	1.22 × 10^0^
Stroke	2.81 × 10^–1^	2.67 × 10^–1^	1.59 × 10^–1^	4.13 × 10^–1^	2.54 × 10^–1^	1.07 × 10^–4^	7.94 × 10^–1^
Total	2.00 × 10^0^	2.00 × 10^0^	1.47 × 10^0^	2.75 × 10^0^	1.28 × 10^0^	7.25 × 10^–2^	4.67 × 10^0^
CHIP-modified background (males)							
BFO	7.92 × 10^–1^	3.06 × 10^–1^	1.14 × 10^–1^	1.44 × 10^0^	1.32 × 10^0^	9.74 × 10^–2^	3.41 × 10^0^
CHD	7.38 × 10^–1^	7.10 × 10^–1^	4.60 × 10^–1^	1.06 × 10^0^	5.98 × 10^–1^	3.01 × 10^–2^	1.96 × 10^0^
Stroke	5.34 × 10^–1^	4.99 × 10^–1^	2.83 × 10^–1^	8.24 × 10^–1^	5.41 × 10^–1^	1.43 × 10^–4^	1.63 × 10^0^
Total	2.93 × 10^0^	3.01 × 10^0^	2.08 × 10^0^	4.32 × 10^0^	2.24 × 10^0^	5.72 × 10^–2^	7.68 × 10^0^
Unmodified background (females)							
HC	8.94 × 10^–2^	8.57 × 10^–2^	5.97 × 10^–2^	1.24 × 10^–1^	6.38 × 10^–2^	6.39 × 10^–3^	2.19 × 10^–1^
CHD	3.68 × 10^–1^	3.59 × 10^–1^	2.45 × 10^–1^	5.06 × 10^–1^	2.61 × 10^–1^	1.99 × 10^–3^	8.98 × 10^–1^
Stroke	3.50 × 10^–1^	3.33 × 10^–1^	1.96 × 10^–1^	5.22 × 10^–1^	3.26 × 10^–1^	1.91 × 10^–4^	1.01 × 10^0^
Total	2.45 × 10^0^	2.41 × 10^0^	1.69 × 10^0^	3.42 × 10^0^	1.73E × 10^0^	6.27 × 10^–2^	6.02 × 10^0^
CHIP-modified background (females)							
HC	5.32 × 10^–1^	2.11 × 10^–1^	6.89 × 10^–2^	9.90 × 10^–1^	9.21 × 10^–1^	5.94 × 10^–3^	2.37 × 10^0^
CHD	5.41 × 10^–1^	5.20 × 10^–1^	3.42 × 10^–1^	7.77 × 10^–1^	4.36 × 10^–1^	3.19 × 10^–3^	1.43 × 10^0^
Stroke	6.71 × 10^–1^	6.24 × 10^–1^	3.54 × 10^–1^	1.03 × 10^0^	6.76 × 10^–1^	3.58 × 10^–4^	2.04 × 10^0^
Total	3.10 × 10^0^	3.24 × 10^0^	2.24 × 10^0^	4.54 × 10^0^	2.30 × 10^0^	1.90 × 10^–1^	7.99 × 10^0^

Hematologic cancer (HC), coronary heart disease (CHD), stroke, and solid cancer point estimate (PE), median, first quartile (Q1), third quartile (Q3), inner quartile range (IQR), lower whisker (LW), and upper whisker (UW) of the lifetime REID% distribution for males. Uncertainty propagation was implemented with a Monte Carlo sampling size of *N* = 100,000. Point estimates of the REID are found by using the most likely values of uncertainty distribution parameters.

Thus far, the analyses have focused on relative percent increases of CHIP affected tissues and the total REID%. The total REID% in this work includes contributions from cancer, CHD, and stroke; however, the NASA PELs are limited solely to 3% mean REID for cancer mortality. Therefore, exposure for this Mars DRM does not exceed the current NASA PELs, although exposures for longer missions are expected to approach or exceed this limit.

## Discussion

Prevention is the primary goal of spaceflight health risk management. Medical tests are required for crew selection and to maintain flight status since certain disease states are considered high risk for the spaceflight environment and are screened out during candidate selection. Unfortunately, not all health issues can be predicted by screening. Additional health risks are removed or reduced via engineering efforts such as providing increased shielding from radiation in the sleep quarters^39^. Extensive planning is required to establish effective medical capabilities and to develop spaceflight medical systems that may be needed for responding to health issues as they occur^71^.

Several risk tools are utilized to better inform the medical management of spaceflight health risk. For instance, the Framingham risk score and Astronaut Cardiovascular Health and Risk Modification (Astro-CHARM) calculator are used to evaluate cardiovascular risk for crew members^15,21,24^. Such calculators provide additional data to better advise flight surgeons regarding flight status decisions but currently do not include the impact of space radiation, although efforts to include it are ongoing^15^. Such models typically include multiple risk factors. For example, the Astro-Charm model predicts the 10-year risk of atherosclerotic cardiovascular disease based on age, sex, systolic blood pressure, total and high-density lipoprotein cholesterol, smoking, diabetes, hypertension treatment, family history of myocardial infarction, high-sensitivity c-reactive protein, and coronary artery calcium scores using a proportional hazard model. The use of these models provides a more accurate reflection of an individual’s background disease risk compared to the use of population average disease rates. Furthermore, these risk calculators can be used to determine the benefits of various interventions such as use of statins, diet, and exercise to reduce body mass index, and are included in guidelines for the primary prevention of cardiovascular disease^20^. However, there remains a subset of individuals—those who do not have alterations in identified risk factors yet still go on to develop cardiovascular diseases— for whom it is not currently possible to predict the development of CVD. In these individuals, the inclusion of additional, emerging, independent risk factors such as CHIP may be an important component of risk assessment.

Improved risk models would allow for more robust calculations of individual risk to a crew member for a specific mission, aid in informed consent conversations with the crew member, and assist in programmatic decisions on the best approaches to control the risks. These same models can be used during missions to inform decisions to extend mission length and the ramifications on crew health. Post-flight, improved risk models would facilitate communication with crew members as to the long-term implications of their actual radiation dose received^15^. Historically, many risk models focus on the near-term risk in the next 5–10 years as the near-term risk is most operationally relevant. Short-term modeling remains useful, but there is room to better communicate the long-term risk to the crew member. Communicating long-term risk is particularly relevant in the case of radiation-induced health risk as many outcomes such as malignancy may take years to develop.

In the present study, increases in the lifetime REID% associated with space radiation exposure on a Mars mission scenario were observed that are directly attributable to the elevated CHD, stroke, and HC risks observed for CHIP carriers. The 16-month Mars design reference mission presented herein represents only one of many potential mission scenarios. Many design reference missions include total mission durations that are approximately 3 years, which increases radiation exposure and associated health risks^45^. Although, historically, the mean age of first flight for crew members is 41 years^37^, future missions to Mars may include a more diverse astronaut age cohort. Projections of REID are highly sensitive to the radiation exposure age for most tissues in the NSCR^60,64^, and the expected excess risk of radiation-induced mortality must be balanced against the mission challenges and requirements.

The current study assumes that added disease burden attributable to CHIP carrier status^26^ applies to population average risk for radiation exposure, which is the current method for assessing morbidity and mortality risks associated with space radiation exposure in the NSCR assessment model. However, it is known that risk profiles in CHIP carriers can also vary depending on a variety of factors not included in the current model including the number of mutations, the specific driver mutations, and the sizes of the variant allele fractions^72–74^. In fact, increased risk for development of de novo AML was found to be associated with clonal hematopoiesis detected years before diagnosis, with the highest risk in individuals harboring mutations in *DNMT3A*, *TET2*, *SF3B1*, *SRSF2*, *TP53*, *JAK2*, and the epigenetic modifiers *IDH1* and *IDH2*, at VAF >0.5–1%^74–76^. The influence of other modifiable and non-modifiable risk factors on CHIP-associated health risks are also coming to light. There is evidence that CHIP prevalence and clonal dynamics are modified by heritable and acquired risk factors including body mass index and lifestyle choices such as diet, tobacco use, history of previous infection, and exposure to cytotoxic therapies^77–80^. For example, enrichment of clones with mutations in the *ASXL1* gene are associated with tobacco use, while radiotherapy and chemotherapy are shown to alter dynamics of clonal expansion with enrichment for mutations in genes for DNA damage response pathway regulators such as *PPM1D*, *CHEK2*, and *TP53*^35,81–86^. Cytotoxic therapies also increase likelihood that a CHIP carrier will develop a secondary therapy-related hematologic malignancy^35,85^. Future modeling efforts and more extensive data would be needed to include these factors in space radiation risk assessments.

Direct evidence for the involvement of CHIP in disease risks in the atomic bomb LSS cohort or other occupational or accidentally exposed populations is, to the best of our knowledge, not currently available, although it has been hypothesized that population risk for radiation-induced leukemia can be attributed to the small fraction of individuals who carry pre-leukemic clones^87^. Genomic changes associated with myelodysplastic syndrome, a precursor to leukemia, have been evaluated in atomic bomb survivors^88^ and suggest a potential contribution of clonal hematopoiesis. Interestingly an association between indoor radon exposure and risk of CHIP is seen in post-menopausal women as part of the Women’s Health Initiative^89^. Further analysis of these and other exposed populations are of interest. As mentioned above, mutations in the tumor suppressor and DNA damage response pathway gene *TP53* are associated with both de novo and therapy-related myeloid malignancies and are considered high-risk mutations^35,85^. Competition studies using bone marrow chimeric mouse models show preferential expansion of *TP53*-deficient clones following low-LET radiation exposure, with no growth advantage in the absence of this selective pressure. This observation points to the role of radiation in cancer promotion vs. initiation in this context^90,91^. It will be important for space applications research to address how intermediate dose, chronic radiation exposure and non-radiation spaceflight stressors may impact CHIP development, clonal dynamics, and associated disease development. Uncovering whether there are specific CHIP mutational profiles that provide a particular fitness advantage in the spaceflight environment may prove useful for risk stratification in astronauts^92^. Competition studies using experimental conditions replicating spaceflight stressors could provide valuable evidence.

The unique baseline risks of hematologic cancer and CVD for CHIP carriers, a common aging-related condition, and the associated increase in the lifetime REID as compared to non-CHIP carriers underscores the need to incorporate personalized risk profiles into space radiation risk assessment. In addition, screening for known risk factors such as CHIP would enable flight surgeons and crew members to develop treatment plans to mitigate risks that may be initiated pre-flight and continue throughout the mission. Post-flight medical monitoring of unique individual risks that have been projected from radiation risk models would also augment routine medical screening of crew.

Despite these advances, the description of CHIP as a clinical entity and emerging risk factor is still fairly new, and approaches for incorporating CHIP carrier status into diagnostic and clinical guidelines are not yet available^73,74,81,93,94^. However, the field is quickly expanding and information on molecular mechanisms and pathways associated with CVD and other CHIP-associated diseases are rapidly evolving. Potential therapeutic approaches are under evaluation^72,93,95,96^. For example, evidence links key CHIP driver mutations to altered immune function associated with CVD, which brings chronic inflammation into light as a putative cardiovascular disease mechanism in CHIP carriers^33^. Treatment options using anti-inflammatory drugs may target enhanced inflammation in these individuals.

In this article, we show that astronaut CHIP carriers are at much greater excess risk of death from radiation exposure than the average U.S. background never-smoker population for a Mars mission scenario when the following assumptions are made. Baseline risks of HC, CHD, and stroke expressed as hazard ratios are incorporated into the NSCR by modification of the background mortality rates. As mentioned in the introduction, this can be interpreted as transferring the risk to a subpopulation of CHIP carriers. There are no CHIP-specific modifications to the dose–response model (linear no-threshold), radiation quality factor, dose rate effects, or the transfer models themselves (i.e., the proportion of EAR versus ERR) or the dose–response model. A specific issue regarding the transfer model is the possible differences in CHIP prevalence in the World War II Japanese atomic bomb cohort from which the radiation risk coefficients are derived and the modern astronaut population. However, current epidemiological evidence shows that leukemia risk is consistent across radiation-exposed cohorts from different populations. For example, radiation-associated leukemia risks are similar in the Japanese-based LSS cohort and Western population-based INWORKS study cohorts^97^, which suggests that any possible differences in CHIP status between these cohorts do not appear to affect the overall magnitude of the leukemia risks. Likewise, specific data on the impact of radiation exposure on disease hazard rates in CHIP carriers is not available, and therefore the approximation used in the current work does not explicitly address this. Future work should include the possibility of such CHIP-specific modifications, but new experimental or epidemiology data would be required. In particular, information on how radiation influences the mutation rates and progression of CHIP clones including dose, dose rate, and quality modification is needed.

Large increases in the HC, CHD, stroke, and total REID% are shown as a function of age after radiation exposure for male and female crew members. Male CHIP carriers are shown to have a larger combined excess radiation risk from HC, CHD, and stroke than from all solid cancers throughout their lifetimes. A similar trend is observed for females; however, the combined risks from HC, CHD, and stroke became larger than solid cancer risks later in life as compared to males. While there are large uncertainties in this study that would require new data to resolve, the need for individualized risk profiles that may be used for medical management and mitigation of spaceflight hazards from radiation exposure is highlighted. Before such risk estimates are suitable for health management decisions, new data and model improvements would be required.

### Supplementary information

Description of Additional Supplementary Files

Supplementary Data 1

Supplementary Data 2

Supplementary Data 3

Reporting Summary

## Data Availability

All data that support the findings of this study are found in the manuscript and supplementary file. Source data for the Figs. 2–4 are available as Supplementary Data 1–3. Monte Carlo distributions associated with the propagation of statistical uncertainty in model parameters are available from the corresponding author upon reasonable request. Published CHIP hazard rates used in this study and associated datasets are described in ref. ^26^.

## Code availability

The HZETRN2020 and NASA Space Cancer Risk model (NSCR) codes used for this study are found at https://software.nasa.gov. In addition, NASA maintains the On-Line Tool for Assessment of Radiation in Space (OLTARIS, available at https://oltaris.nasa.gov), where users may request an account to perform radiation transport and projections of the REID with the NSCR model by using a simple graphical user interface.
