# Supplementary material for: Considering clonal hematopoiesis of indeterminate potential in space radiation risk analysis for hematologic cancers and cardiovascular disease
Source: Commun Med (Lond). 2024 Jun 11;4:105. doi: 10.1038/s43856-023-00408-4 (PMC11166645; doi:10.1038/s43856-023-00408-4)
Supplement: Supplementary file 1 — Description of Additional Supplementary Files [file 43856_2023_408_MOESM1_ESM.pdf]

Description of Additional Supplementary Files

Considering Clonal Hematopoiesis of Indeterminate Potential in Space Radiation Risk Analysis for Hematologic Cancers and Cardiovascular Disease

Charles M. Werneth, Zarana S. Patel, Moriah S. Thompson, Steve R. Blattnig, Janice L. Huff

File name: Supplementary Data 1

Description: Supplementary Data 1 contains source data used to generate Figure 2 along with accompanying descriptive notes.

File name: Supplementary Data 2

Description: Supplementary Data 2 contains source data used to generate Figure 3 along with accompanying descriptive notes.

File name: Supplementary Data 3

Description: Supplementary Data 3 contains source data used to generate Figure 4 along with accompanying descriptive notes.
