# Supplementary material for: Considering clonal hematopoiesis of indeterminate potential in space radiation risk analysis for hematologic cancers and cardiovascular disease
Source: Commun Med (Lond). 2024 Jun 11;4:105. doi: 10.1038/s43856-023-00408-4 (PMC11166645; doi:10.1038/s43856-023-00408-4)
Supplement: Supplementary file 5 — Reporting Summary [file 43856_2023_408_MOESM5_ESM.pdf]

nature portfolio

Corresponding author(s): Charles M Werneth

Last updated by author(s): May 16, 2023

Reporting Summary

Nature Portfolio wishes to improve the reproducibility of the work that we publish. This form provides structure for consistency and transparency in reporting. For further information on Nature Portfolio policies, see our Editorial Policies and the Editorial Policy Checklist.

Statistics

For all statistical analyses, confirm that the following items are present in the figure legend, table legend, main text, or Methods section.

n/a	Confirmed	
☐	☒	The exact sample size (*n*) for each experimental group/condition, given as a discrete number and unit of measurement
☐	☒	A statement on whether measurements were taken from distinct samples or whether the same sample was measured repeatedly
☒	☐	The statistical test(s) used AND whether they are one- or two-sided<br>*Only common tests should be described solely by name; describe more complex techniques in the Methods section.*
☒	☐	A description of all covariates tested
☐	☒	A description of any assumptions or corrections, such as tests of normality and adjustment for multiple comparisons
☐	☒	A full description of the statistical parameters including central tendency (e.g. means) or other basic estimates (e.g. regression coefficient) AND variation (e.g. standard deviation) or associated estimates of uncertainty (e.g. confidence intervals)
☒	☐	For null hypothesis testing, the test statistic (e.g. *F*, *t*, *r*) with confidence intervals, effect sizes, degrees of freedom and *P* value noted<br>*Give P values as exact values whenever suitable.*
☒	☐	For Bayesian analysis, information on the choice of priors and Markov chain Monte Carlo settings
☒	☐	For hierarchical and complex designs, identification of the appropriate level for tests and full reporting of outcomes
☒	☐	Estimates of effect sizes (e.g. Cohen's *d*, Pearson's *r*), indicating how they were calculated

Our web collection on statistics for biologists contains articles on many of the points above.

Software and code

Policy information about availability of computer code

Data collection	The HZETRN2020 and NSCR codes used for this study are found on https://software.nasa.gov. Characterization of the space radiation environment, spacecraft geometry and composition, and radiation transport were performed with the HZETRN2020 code, and the risk of exposure induced death was evaluated with the NSCR code.
Data analysis	Data analysis and visualization were performed with python-3.9.10.

For manuscripts utilizing custom algorithms or software that are central to the research but not yet described in published literature, software must be made available to editors and reviewers. We strongly encourage code deposition in a community repository (e.g. GitHub). See the Nature Portfolio guidelines for submitting code & software for further information.

Data

Policy information about availability of data

All manuscripts must include a data availability statement. This statement should provide the following information, where applicable:

- Accession codes, unique identifiers, or web links for publicly available datasets
- A description of any restrictions on data availability
- For clinical datasets or third party data, please ensure that the statement adheres to our policy

All data that supports the findings of this study are found in the manuscript and supplementary file. Source data for the figs. 2-4 are available as Supplementary Data 1-3. Monte Carlo distributions associated with the propagation of statistical uncertainty in model parameters are available from the corresponding author

upon reasonable request. Published CHIP hazard ratios used in this study and associated datasets are described in Jaiswal et al. N Engl J Med 371, 2488–2498 (2014).

Human research participants

Policy information about studies involving human research participants and Sex and Gender in Research.

Reporting on sex and gender	N/A<br><br>Hazard rates from a published human cohort study (Jaiswal et al.) were used in our analysis. The published hazard ratio data were then used to scale published disease rates of the average US background population in the NASA Space Cancer Risk model. All details of the NASA Space Cancer Risk Model are fully described in Cucinotta et al. Excess radiation risks for males and females were studied in our analysis.<br><br>Cucinotta, F. A., Kim, M.-H. Y. & Chappell, L. J. Space radiation cancer risk projections and uncertainties – 2012; NASA TP-2013-217375. (2013).<br><br>Jaiswal, S. et al. Age-related clonal hematopoiesis associated with adverse outcomes. N Engl J Med 371, 2488–2498 (2014).
Population characteristics	N/A. See discussion above.
Recruitment	N/A. See discussion above.
Ethics oversight	N/A. See discussion above.

Note that full information on the approval of the study protocol must also be provided in the manuscript.

Field-specific reporting

Please select the one below that is the best fit for your research. If you are not sure, read the appropriate sections before making your selection.

☒ Life sciences ☐ Behavioural & social sciences ☐ Ecological, evolutionary & environmental sciences

For a reference copy of the document with all sections, see nature.com/documents/nr-reporting-summary-flat.pdf

Life sciences study design

All studies must disclose on these points even when the disclosure is negative.

Sample size	N/A. See discussion in Human Research Participants.
Data exclusions	N/A. See discussion in Human Research Participants.
Replication	N/A. See discussion in Human Research Participants.
Randomization	N/A. See discussion in Human Research Participants.
Blinding	N/A. See discussion in Human Research Participants.

Reporting for specific materials, systems and methods

We require information from authors about some types of materials, experimental systems and methods used in many studies. Here, indicate whether each material, system or method listed is relevant to your study. If you are not sure if a list item applies to your research, read the appropriate section before selecting a response.

March 2021

Materials & experimental systems

n/a	Involved in the study
☒	☐ Antibodies
☒	☐ Eukaryotic cell lines
☒	☐ Palaeontology and archaeology
☒	☐ Animals and other organisms
☒	☐ Clinical data
☒	☐ Dual use research of concern

Methods

n/a	Involved in the study
☒	☐ ChIP-seq
☒	☐ Flow cytometry
☒	☐ MRI-based neuroimaging
